# Clinico-epidemiology and management of hump-nosed pit viper (*Hypnale* spp.) bites in dogs

**DOI:** 10.1038/s41598-022-12386-z

**Published:** 2022-05-17

**Authors:** Ranjith Adhikari, Lalith Suriyagoda, Amal D. Premarathna, Rando Tuvikene, Chandima Mallawa, Niranjala De Silva, Ashoka Dangolla, Indira Silva, Indika Gawarammana

**Affiliations:** 1 Department of Veterinary Clinical Sciences, Faculty of Veterinary Medicine and Animal Science, University of Peradeniya, Peradeniya, Sri Lanka; 2 Department of Crop Science, Faculty of Agriculture, University of Peradeniya, Peradeniya, Sri Lanka; 3 School of Natural Sciences and Health, Tallinn University, Narva mnt 29, 10120 Tallinn, Estonia; 4 Department of Medicine, Faculty of Medicine, University of Peradeniya, Peradeniya, Sri Lanka

**Keywords:** Biochemistry, Structural biology, Medical research, Risk factors

## Abstract

Human envenoming from the bite of the abundant hump-nosed pit viper (Hypnale *spp.*) (HNPV) is a frequent occurrence with victims experiencing unpleasant and sometimes life-threatening consequences. Further, clinico-pathology, treatment and management measures in HNPV envenomed dogs are under recognized. Prospective investigations were performed to assess the clinico-pathology and management options for HNPV envenomed dogs brought to the University of Peradeniya’s Veterinary Teaching Hospital from January, 2012 to March 2018. We recorded the local and systemic manifestations, hematological and urinary abnormalities of 78 dogs in which HNPV bite had been witnessed by the owner. Mild swelling, extensive swelling, hemorrhagic blistering and hemorrhagic bullae at the site of bite were observed in 59%, 31%, 6% and 4% of the dogs, respectively. Some dogs were subjected to surgical excision of necrotized tissue including limb amputation. We observed the following systemic clinical effects in envenomed dogs: neurotoxicity (13%), acute kidney injury (AKI) (14%) and coagulopathy (16%). All dogs showed leukocytosis with mean white blood cell count of 25.25 × 10^3^/µL. Mild anemia and thrombocytopenia were detected in 29% of the dogs. There was a significant correlation between extent of local tissue injuries with length of hospitalization (LH). The mean time of coagulopathy observed was 21.3 h (IQR: 8–48 h). In coagulopathic dogs, there was a strong correlation between LH and extent of local tissue injury (*r*_*s*_ = 0.7751, *P* < 0.0001); LH and whole blood clotting time(CT) (*rs* = 1.0, *P* < 0.0001); PT and aPTT (*r*_*s*_ = 0.4712, *P* < 0.001). LH was significantly correlated with the development of AKI (*p* = 0.0013). Lack of specific antivenom (AVS) for HNPV envenoming provided an opportunity to study the remaining treatment options. Therefore, the study allowed the identification of local and systemic effects, hematological abnormalities, possible supportive treatments and drawbacks of management measures for envenomed dogs.

## Introduction

Hump nosed pit-vipers (HNPV) of the genus *Hypnale* are the smallest of the known pit vipers (< 50 cm in total length) and inhabit Sri Lanka and the Western Ghats mountains of the Indian peninsula^1,2^. Historically, there has been controversy and disagreement about which species should be included in the genus *Hypnale*. According to the taxonomy established by Fitzinger in 1843, the Hump-nosed pit viper is represented in the genus *Hypnale.* Consequently, three species that belong to Genus *Hypnale* have been identified namely, *Hypnale hypnale*, *Hypnale nepa* and *Hypnale zara. H. hypnale* is widely distributed across the island, while *H. nepa* can be found in the Central Highlands and *H.zara* in the South Western wet zone low lands^3^. Further*, H. nepa* and *H.zara* are endemic to Sri Lanka^2,3^. Forest and anthropogenic environments are the commonest habitats of *H. hypnale* and *H. nepa,* but *H. zara* is restricted to lowland rainforests^3^.

Although human fatalities from HNPV bite are infrequent they occur across a widely dispersed area in Sri Lanka and South-Western India, a high incidence being reported^1,3–7^. However, HNPV envenoming has a low comparative incidence in dogs^8^. *H. hypnale* is one of the first snakes for which the pathophysiology of envenoming was scientifically studied. Davy in 1821 reported local swelling, deep ulceration and bloody stool resulting from envenoming in dogs, chickens and frogs^9^.

*Hypnale* spp*.* venoms demonstrated severe cytotoxicity by inhibiting cell proliferation in invitro in chick biventer cervicis preparations and confirmed lesser neurotoxicity, myotoxicity and procoagulant activity^10^. Further, in this study, *Hypnale* spp venoms exhibited low IC_50_ values compared to cytotoxic *Naja mossambica* and Australasian death adders indicating that the venom contains cytotoxins of greater potentcy compared with other snake venoms. Proximal tubular cell injury and acute tubular necrosis with intact basement membrane have been observed in mouse models indicating possible direct nephrotoxicity following HNPV envenoming^11^.

Predominately HNPV bites in humans cause local effects such as pain and swelling, local hemorrhagic blistering, regional lymphadenopathy and rarely severe local necrosis and gangrene^2,6,12,13^ but 10% of victims suffer kidney injury^14^ and death following acute kidney injury has been documented in man^15,16^. According to the recent studies conducted^17^ *Hypnale* spp*.* is considered as a highly venomous snake which needs antivenom as death have been reported in man following HNPVs bite. Unlike the bites from other species of venomous snakes, the occurrences of systemic effects are rare and unpredictable with HNPV bites^2^. The rarity and unpredictability of the sporadic occurrence of these potentially fatal systemic effects of envenoming by HNPV have made it an important species for investigation^2,18^. Moreover, the presence of nephrotoxic properties has been reported from *H. hypnale*, and from *H. zara*, but the nephrotoxic effect from *H. nepa* bite is as yet unknown^11,15^. Cardiac involvement has been recorded with transient ECG changes but evidence of myocardial trauma has not been proven due to normal levels of cardiac troponin in HNPV envenoming in humans^1,19^. However, HNPV venom has been shown to cause lung pathology^1,10^. In Sri Lanka, the Indian polyvalent antivenom (AVS) currently in use to treat venomous snake bites in man does not include HNPV antivenom. In addition, a venom quantification study was able to demonstrated venom antigen in circulation up to 24 h post bite in human due to scarcity of specific antivenom for *Hypnale* spp. envenoming^20^.

Treatment protocols using AVS for Naja *naja* and *Daboia russelii* envenoming in dogs have been established recently^21,22^. However, detailed investigations beyond several fundamental studies related to HNPV bite envenoming are scarce. The benefit of early surgical intervention to minimize the extent of local tissue injury due to *H. hypnale* envenoming in dogs has been suggested in one study^23^. However, the consequences of HNPV in dogs has not been studied in depth, and venom concentration has not been measured. Hence, there is currently a paucity of clinical profiles, treatment and management plans for dogs in Sri Lanka. This study utilised HNPV envenomed dogs to investigate the role of HNPV venom in the observed clinical profile and biochemical changes in body fluids. Crucially, these studies provide evidence of a hitherto unidentified action of *H. hypnale* venom.

## Materials and methods

### Study setting and clinical examination of patients

Dogs that presented to the emergency service of the Veterinary Teaching Hospital (VTH) of the University of Peradeniya following hump-nosed pit viper (*Hypnale hypnale*) envenoming were studied prospectively from January 1, 2012, to March 31, 2018. The study was conducted in accordance with relevant guidelines and regulations. Three methods were used to recognize offending snakes viz visual identification of snake specimens brought by dog owners;^22,23^; identification through photographs of the culprit snakes taken on mobile phones by owners and, identification by owners through photographs and preserved specimens of the culprit snakes. All the clinical examination procedures were performed in the intensive care unit of the VTH. The study was conducted after the informed consent was given by the dog owners. In order to find out the modality of HNPV envenoming detailed clinical examinations were performed on all patients. All the body systems were examined and qualitative and quantitative measurements of the clinical signs and symptoms were recorded. In order to classify the extent of tissue injury/s at the site/s of bite the grading system explained in the studies conducted by Grassi et al., 2016 and Adhikari et al., 2020 were used in this study^21,24^. Accordingly, severity of local tissue reactions at the site of bite of the patients were graded.

### Laboratory investigations

On admission, blood and urine samples were collected for hematological, biochemical tests and urinalysis respectively, which were repeated until they normalized. Blood samples were collected by venipuncture (cephalic vein, saphenous vein or sometimes jugular vein) from all the patients (*n* = 78) before initiation of treatment. 2.5 mL of blood was placed in a plastic tube containing Ethylenediamine tetraacetic acid (EDTA) 1.5–2 mg/mL as the anticoagulant to perform full blood count (FBC), alanine aminotransferase (ALT), total protein (TP), aspartate aminotransferase (AST), Albumin, fibrinogen Blood Urea Nitrogen (BUN) and creatinine values. The same tests were repeated 24 h after the initiation of supportive treatments. A volume of 0.9 mL of blood was collected into a tube containing 3.2% *tri*-sodium citrate at a ratio of 9:1 to perform Partial Thromboplastin Time (PT) and Activated Partial Thromboplastin Time (aPTT). The sample was then centrifuged at 2000 g for 10 min, and plasma was separated. The semi-automated biochemistry analyser (ERBA Chem-7, Germany) was used to perform PT and aPTT tests. About 1 mL of blood was placed in a Khan tube to measure the clotting time (CT). Moreover, the exact test is termed as whole blood clotting time (WBCT20) in human medicine^4^.

### Clinical care

Each case was considered as an emergency; therefore, the dogs received care in the Intensive Care Unit where IV access was established. As supportive treatment hydrocortisone (Hydrocortisone sodium Succinate for injection BP 100 mg, AMN Life Science Pvt Ltd, India; 10 mg/kg, IV, q.i.d.), chlorpheniramine maleate (Allervit 1% W/V,1 mL, Healthcare Pvt. Ltd, India) 0.4 mg/kg, IV, q.i.d.), cloxacillin sodium (for Injection BP 250 mg, Vysali Pharmaceuticals Limited, India; 20 mg/Kg, IV, b.i.d.), metronidazole (Metronidazole Intravenous Infusion BP 500 mg/100 mL, Claris Life Sciences Limited, India; 20 mg/Kg, IV, b.i.d.), and fluids (normal saline, Lactated Ringer’s Solution, 10% Dextrose in appropriate volumes) were administered intravenously for the initial 24 h of hospitalization. The treatments were continued until the patients recovered. The currently available AVS does not include antibodies for the venom of HNPV. Therefore, none of the patients were treated with AVS.

#### Medical and surgical management of hemorrhagic blistering and necrosis

**Figure 1:**
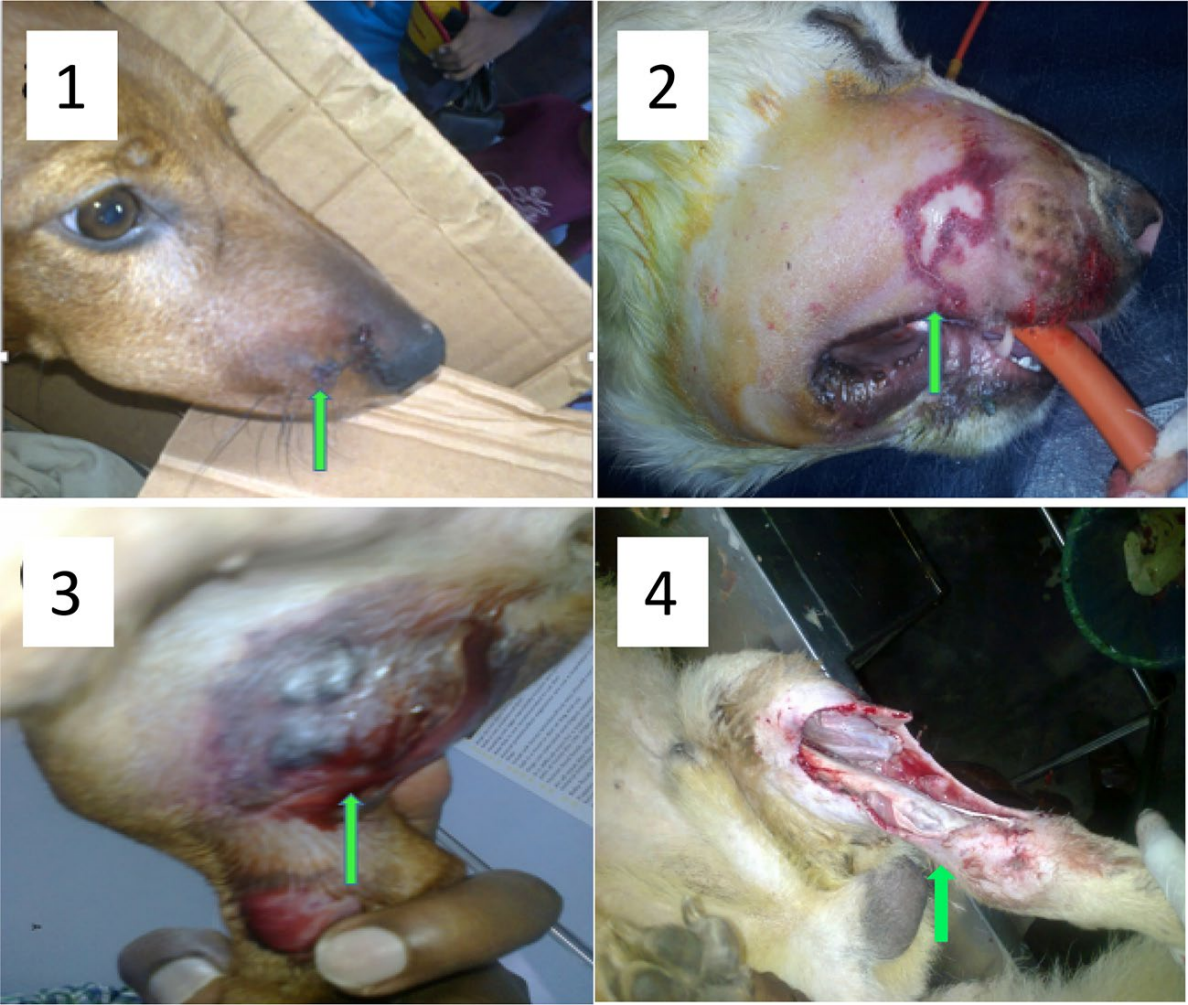
Local tissue reactions observed due to hump-nosed pit viper bite (arrows indicate the site of fang marks/lesions). (**1**) Mild swelling, (**2**) Extensive swelling, (**3**) Hemorrhagic blistering, (**4**) Ruptured hemorrhagic bullae.

Wounds with necrotic tissue, and heamorrhagic blisters, when observed, were subjected to treatment with the aim of reducing the amount of necrotic tissue and further aggravation of necrosis. The necrosed tissue was surgically excised at the site of bite under general anesthesia. Supportive treatment was initiated before such surgical intervention and intravenous isotonic saline was continued in order to stabilize the patients for general anesthesia. Diazepam (Calmvita 5 mg/mL, Healthcare Pvt. Ltd, India; 0.5 mg/kg, IV, stat.) was given as anaesthetic pre medication. Propofol; (Anesia™ 1% Injection for Intravenous Infusion 50 mL /Vial, Claris Lifesciences Limited, Ahmedabad, India; 4 mg/kg, IV), an ultra-short-acting compound was given to induce general anesthesia and maintained throughout the procedure using propofol at the rate of 0.1 mg/kg/min. The surgical site was prepared using isopropyl alcohol, chlorhexidine and povidone iodine, in a standard manner. Surgical excision of the blistered area with necrotized tissue at the edges of the wound area was performed. Myotomy was performed and devitalized tissue were removed as required. Muscles and subcutaneous tissues were sutured using 2/0 chromic catgut and skin was sutured using suture nylon, applying the simple interrupted suture pattern. The same antibiotic combinations were continued until completion of wound healing. On examination, a hemorrhagic bullae was observed in the left medial entire tibia-fibula region and the bullae has ruptured exposing the medial aspect of the entire shaft of both tibia and fibula (Fig. 1). Initially, the wound was cleaned using hydrogen peroxide and necrotized tissues were excised. Mid femoral amputation was performed under general anaesthesia.

#### Management of patients with coagulopathy

Detection of elevated PT, aPTT, and CT were measured in dogs with coagulopathy and were kept with restricted movements to prevent further aggravation of the bleeding tendency. Coagulation tests were repeated at 2 h intervals in the first 12 h of admission and then 6 hourlies till normal results were obtained. Patients were given IV infusion of isotonic saline to ensure adequate urine output. The decision to discharge patients from the hospital was taken upon improvement of the clinical condition. The dogs’ activities were restricted during the period of hospitalization.

#### Management of acute kidney injury due to hump-nosed pit viper envenoming

If signs of acute kidney injury (AKI) were observed isotonic saline (0.9%) was administered by intravenous infusion to ensure adequate urine output. Patients were observed for the production of urine and if anuric or oliguric, furosemide (furosemide 20 mg/2 mL solution for injection, steril-Gene Life Sciences (P) Ltd, India; 2 mg/kg IV, 4 mg/kg or 6 mg/kg IV) was administered and observed for one hour to determine the production of urine. If there was no diuretic effect, then furosemide was repeated at the dose rate of 4 mg/kg IV.

### Statistical analysis

Data management and descriptive statistics were performed using MINITAB 16 software and Microsoft Excel (Excel 2010). The Spearman’s rank correlation was used to find relationships between variables (i.e., time lapse, hospital stay, extent of injuries, PT, APTT, CT, fibrinogen). Comparisons between groups (e.g., ‘presence’ or ‘absence’ of kidney injuries, and ‘yes’ or ‘no’ for coagulopathy) for time lapse and the duration of hospital stay as dependant variables were done using Wilcoxon Rank Sum test. Comparisons among the groups (e.g., extent of injuries (1, 2, 3, 4) on the time lapse and the duration of hospital stay as dependant variables were done using Kruskal–Wallis test. All the interpretations (i.e., significances) were made at the α = 0.05, unless stated.

### Ethics approval

Ethical clearance was obtained from the Ethical Review Committee of the Faculty of Veterinary Medicine and Animal Science, University of Peradeniya, Sri Lanka (Ref No.VER-14–012, 17 December 2014), which is on par with the international standards of ethics on animal experimentation. All experimental procedures and animal care were approved by the Faculty Ethics Committee, Faculty of Veterinary Medicine and Animal Science, University of Peradeniya, Sri Lanka.

### Consent for publication

We certify this manuscript has not been published elsewhere and is not submitted to another Journal. All authors have approved the manuscript and agreed with submission to Journal of Veterinary Research.

## Results

There were 78 patients reported during the study and 68 of them were males (87%), and 66 (85%) were below the age of one year. The median age was 6.5 months and IQR was 4–10.25 months. The majority of bites *n* = 61 (78%) were reported during 0500–0700 h and 1700–2000 h.

### Local clinical manifestations of hump-nosed pit viper envenoming

All the patients exhibited signs of severe pain upon palpation of bitten area. The main sites of fang marks were the face (rostrum, mandible, lips) and neck regions *n* = 68 (87%). Fang marks were observed in medial aspect of hind limbs in *n* = 11 (14%) of dogs. Mild swelling, extensive swelling, hemorrhagic blistering and hemorrhagic bullae were observed in these patients. If the affected anatomical site was swollen to an additional half of its normal size, it was recorded as extensive swelling. The length of hospitalization was increased with the progression of tissue injuries (*p* < 0.05, Table 1, Fig. 1).

**Table 1:** Extent of tissue trauma at bite site of dogs.

Extent of local tissue reaction at bite site	Number of patients	Percentage of dogs (%)	Mean length of hospitalization (days)	IQR (days)
1. Mild swelling	46	59	3.8	2.4–5
2. Extensive swelling	24	31	10.2	9–12
3. Hemorrhagic blistering	5	6	18.2	11.5–24
4. Hemorrhagic bullae	3	4	28.0	25–33

*IQR* Interquartile Range.

Dogs with hemorrhagic blistering at the site of bite (n = 5, 7%) was subjected to surgical excision of necrotic tissue by fasciotomy and myotomy under general anesthesia to minimize further aggravation from necrosis. In n = 1 (0.78%) patient with a ruptured heamorrhagic bullae and subsequent exposure of the shaft of the medial side of the left tibia fibula, mid femoral amputation was required.

### Systemic manifestations of hump-nosed pit viper envenoming

Systemic manifestations of neurotoxicity, hypersalivation, hyperesthesia and dilated pupils, were observed in 10 dogs (13%). The demographic and coagulation parameters of dogs with HNPV bite envenoming are presented in Table 2.

**Table 2:** Demographic and clotting parameters variation of dogs with HNV bite envenoming.

Variable	Entire group of dogs (*N* = 78)		Coagulopathic dogs (*N* = 16)		Non-coagulopathic dogs (*N* = 62)	
	Mean	Std Err	Mean	Std Err	Mean	Std Err
Time-lapse (hours)	6.0	1.02	8.0	4.5	5.5	0.81
Hospital stay (days)	7.4	0.71	11.3	1.77	6.4	0.74
PT (seconds) (Normal range 7–11)	24.0	4.19	81.7	13.15	9.1	0.15
aPTT (seconds) (Normal range 11–22)	30.6	3.85	83.7	11.55	16.9	0.52
CT (minutes) (Normal range3-12.5)	8.0	0.54	23.6	1.75	7.2	0.38
Fibrinogen (mg/dL) (Normal range 200–400)	264.0	9.84	135.9	8.95	301.9	7.02

(*Std Err*. Standard Error).

Coagulopathy was suggested in 16 dogs (21%) with evidence of prolonged PT, aPTT, CT and hypofibrinogenemia; although bleeding from the bite site or injection site was not observed. The mean time required for development of coagulopathy was 21.3 h (IQR: 8–48 h). Moreover, the mean time lapse from bite, LH, PT, aPTT and CT were higher in dogs with coagulopathy than in the non-coagulopathic dog group. The mean LH of dogs with coagulopathy was greater (11.3 days) than that of non-coagulopathic dogs (6.6 days). The lowest fibrinogen concentration was observed in the coagulopathic dog group (Table 2).

For coagulopathic dogs, correlations between the time lapse, LH, extent of injury, PT, aPTT, CT and fibrinogen were not significant (Table 3), except in few instances, *i.e*., positive correlations between LH and extent of local tissue injury (*r*_*s*_ = 0.78, *p* < 0 0.0001); length of hospitalization and CT (*r*_*s*_ = 1.0, *p* < 0.0001); PT and aPTT (*r*_*s*_ = 0.47, *p* < 0 0.001). In non-coagulopathic dogs, correlation between fibrinogen concentration and aPTT was significant (*r*_*s*_ = 0.46303, *p* = 0.0002) while other variables were not correlated.

**Table 3:** Spearman’s rank correlation coefficient (*r*_*s*_) between the tested variables from coagulopathic (above diagonal) and non-coagulopathic (below diagonal) dogs.

	Time lapse (hours)	Length of hospitalization (days)	Extent of injury	PT (seconds)	aPTT (seconds)	CT (minutes)	Fibrinogen (mg/dL)
Time lapse (hours)		1.00000 0.34247 16	− 0.0706 0.5392 16	− 0.12772 0.6501 16	− 0.11778 0.6760 16	− 0.5000 0.6667 16	− 0.50324 0.0558 16
Length of hospitalization (days)	0.12244 0.3431 62		0.7751 < .0001 16	− 0.0349 0.8978 16	− 0.2169 0.4196 16	1.0000 < .0001 16	0.2933 0.1643 16
Extent of injury	0.12168 0.3462 62	− 0.14626 0.2566 62		0.0051 0.9850 16	− 0.1608 0.5520 16	0.8660 0.3333 16	0.1702 0.4266 16
PT (seconds)	0.06698 0.6050 62	0.00871 0.9464 62	0.06639 0.6082 62		0.4712 0.0654 16	− 0.5000 0.6667 16	0.0052 0.9846 16
aPTT (seconds)	0.09023 0.4856 62	− 0.07368 0.5693 62	− 0.01474 0.9095 62	1.00000 62		− 0.5000 0.6667 16	− 0.0120 0.9649 16
CT (minutes)	0.14040 0.2761 62	− 0.06238 0.6300 62	− 0.07083 0.5843 62	0.03798 0.7694 62	0.46303 0.0002 62		− 0.5000 0.6667 16
Fibrinogen (mg/dL)	− 0.11006 0.4282 62	0.16904 0.2217 62	0.18795 0.1735 62	0.04359 0.7543 62	0.46303 0.000262	0.11996 0.3876 62	

Three values per each pair of variables indicate the *r*_*s*_, corresponding probability and sample size from top to bottom, respectively.

Nephrotoxicity was observed in 11 dogs (14%). AKI was indicated by oliguria with impaired kidney functions within 24 h after admission (Table 4). Hematuria with isosthenuria were also detected. The mean length of hospitalization of dogs with AKI was 12 days while that of non-AKI dogs was 7 days (*p* = 0.0013). However, dogs with and without kidney injuries had similar time lapse for admission (*p* = 0.374).

**Table 4:** Renal functions of patients, which developed acute kidney injuries (*n* = 11).

Character	Normal range	Median	Minimum	Maximum
Elevated BUN levels	10–28 (mg/dL)	87.5	72	103
Elevated creatinine levels	0.5–1.5 (mg/dL)	2.05	1.8	2.3
Impaired urine output	0.5 (mL/kg/h)	0.34	0.31	0.37

### Abnormalities detected on full blood count (FBC)

Hematological analysis revealed leukocytosis in all the samples. The descriptive statistics of FBC are depicted in Table 5, although ALT, AST and Albumin were observed within normal ranges.

**Table 5:** Abnormalities observed in full blood count in hump-nosed pit viper envenomed dogs.

Character	Number of dogs	% of dogs	Mean	Median	Minimum	Maximum	IQR	Range
Leukocytosis (× 10^3^/mL)	78	100	25.25	24.29	20.03	38.00	4.912	17.97
Anemia (%)	21	29	23.23	23.76	20.12	25.98	3.40	5.80
Thrombocytopenia (× 10^3^/mL)	21	29	127.3	89.0	49.0	198	95.0	149.0

*IQR* Interquartile range.

Except leukocytosis, 48 dogs showed only local clinical manifestations while 30 dogs showed systemic manifestations and/or deviated laboratory test results. There was no significant difference in the time lapse for hospitalization between the dogs with either local and systemic manifestations (*p* = 0.350). However, the length of hospitalization of dogs with local manifestations was shorter (5.3 days) than that of the dogs with systemic manifestations (11.2 days) (*p* < 0.0001). All the patients recovered only with supportive treatments without administration of AVS. However, due to the necrotic effects of HNPV venom 10% of patients suffered permanent disabilities.

## Discussion

This study is the first to highlight the clinical sequelae and laboratory findings recorded during the clinical care and management of dogs envenomed by *Hypnale* spp, one of the commonest venomous snakes in Sri Lanka. Consistent with studies of snakebite incidence from other venomous snakes this study observed a higher proportion of males suffered HNPV bites. It is hypothesised this reflects the more prominent territorial behaviour of male dogs^8,22,23^. This observation parallels the incidence of snakebite in human males in farming communities due to social norms governing occupational roles^4,25^.

The majority of HNPV bite victims were young dogs. It is speculated that younger dogs have a greater tendency than old dogs to sniff peculiar moving objects, although the reason is poorly understood but may be related to the development of recognition and aversion to threats. A study in 2016 drew a connection between this exploratory behaviour and the facilitation of allowing snakes to enter households and consequently heighten risks to humans^26^. Another study indicates dogs may play a significant role as sentinels leading to avoidance of human snakebite^27^. Correspondingly, the economically active age groups are the most susceptible to become HNPV bite victims in man^2,4,25^. Therefore, it is important to note that to identify hotspots of snakebite risk to both human and domestic animals a new concept called “citizen science and action ecology” has emerged based on digital and social innovations^27,28^. Such exploration will help herpetologists to design and implement policies on prevention and treatment of snakebite in humans and animals.

In Sri Lanka *H. hypnale* is largely nocturnal^29^ and the majority of bites recorded in this study occurred during darkness or the crepuscular hours. *H. hypnale* is most commonly found under stones, logs and leaf litter in and around human settlements. Therefore, the pattern of behaviour of both dog and HNPV increases the possibility of envenoming in dogs at dusk and dawn^8^. By contrast the majority of HNPV bites in human are reported in day time in their home gardens^2^. The preference for anthropogenic habitats by HNPV reflects the relationship of bite time and active hours of the day of both man and dog.

Fang marks among the majority of dog victims of *N. naja* and *Dabia russelii*^21,22^ are seen in the head and neck region and a similar bite distribution pattern was seen in the majority of HNPV bitten dogs. Sharp canine teeth have evolved as the major weapons for protection in dogs and they fight facing ahead. Dogs have a highly sensitive of olfaction; the detection and recognition of odorants plays an essential role in many adaptive dog behaviours, such as escape from danger, so they tend to sniff strange objects^30^. Therefore the observation of fang marks around head and neck in the majority of snakebite victims is not unexpected. By comparison the vast majority of fang marks in humans are observed on either the feet or hands^2,4,31^. Failure to use protective measures when entering environments with poor visibility where snakes may dwell is instrumental in acquiring snakebite in man^31^. The education of owners to restrict dogs’ activities, particularly young dogs, during the hours of darkness or twilight should significantly lessen the possibility of becoming a HNPV bite victim.

Our work describes, for the first time, the detection of local clinical signs of mild swelling, extensive swelling, hemorrhagic blistering and hemorrhagic bullae due to the venom of *H. hypnale* in all dogs.

Snake venom metalloproteases are Zinc-dependent metalloproteases and, are classified into four different protease sub classes: P-I, P-II, P-III and P-IV, almost as abundant as phospholipase A2 in *H. hypnale* venom and belong to ‘P-I protease’ subclass, and are another major toxin class of concern for clinico-pathological correlation^32^. The venom metalloprotiases of *H. hypnale* venom have been recognized as capable of inducing local hemorrhage, activate prothrombin and cause fibrin (ogen)olysis^32–35^, and also the venom showed direct thrombin-like enzyme activity on fibrinogen^36^. P-I protease contributes to local tissue symptoms in *H. hypnale* envenoming in 90% of cases of man^32^. The clinical manifestations are inflammation (swelling and pain), wound hemorrhage, blistering, and irreversible tissue destruction i.e. necrosis, and involve disruption of capillary vessel integrity, matrix degradation, dermal–epidermal junction separating and secretion of proinflammatory mediators ie leading to tissue ischemia, disrupted tissue repair, and a varied degree of tissue damages (e.g. dermonecrosis, myonecrosis)^1,4,15,32^. Mild swelling and extensive swelling observed in this study are consistent with the observation of envenoming in dogs due to *N. naja* in Sri Lanka^21^. Further similarity of varying degrees of swelling, haemorrhagic blisters, local tissue necrosis and regional lymphadenopathy are reported in different studies in human victims of HNPV bite envenoming^1–4,15^. Mild and extensive swelling at the site of the bite resolved spontaneously without any adverse outcome as observed in previous studies in both man and animals^21,37^. However, to address snakebite-induced local tissue damage and implement wound care, surgical management, including limb amputation on rare situations, had to be employed for HNPV envenomed dogs, as has been necessary for *N.naja* envenomed dogs^21^. As well as palliative treatment measures, wound care, surgical intervention, skin grafting and occasional limb amputation, are practiced for HNPV envenomed human patients^1^. There is thus, some evidence that early surgical intervention for such dogs is beneficial to minimize tissue necrosis, cost of treatment and to improve quality of life for dogs. However, in addition to the risk of mortality, a venomous bite by a member of Viperidae or Elapidae families also causes a severe local tissue trauma which frequently leads to amputation of the bitten limb to save the life of the victim^38^. Therefore, surviving victims suffer a substantially-reduced quality of life due to the multiple effects of permanent physical and psychological disabilities. Hence, exploring the potential utility of small molecules as community-based therapeutics to prevent local tissue damage following snakebite will help to prevent and minimize the extent of local tissue damage.

The development of systemic manifestations was fewer compared to local clinical signs in patients in this study. Severe systemic envenoming of coagulopathy, AKI and neurotoxicity, were present in only approximately 20% of victims. Interestingly, neurological manifestations were the least common systemic manifestions reported in this study. Moreover, focal neuronal degeneration in brain have been reported in mice models^39^. However, neurotoxicity have not been reported in humans patients^4^. In humans, systemic effects: nephrotoxicity, coagulopathy, thrombocytopenia and spontaneous haemorrhage, as well as less common effects (nausea, vomiting) are less common^6,13,39^. However, despite being rare the unpredictability of sporadic and potentially fatal systemic effects of HNPV envenoming confirms the significance of this species^31^. Similar to the current study, systemic clinical signs of coagulopathy and AKI have been reported, although not frequently, in HNPV bite envenoming in humans^31,40^.

Positive correlations between LH and extent of local tissue injury (rs = 0.78, *p* < 0 0.0001); length of hospitalization and CT (rs = 1.0, *p* < 0.0001); PT and aPTT (rs = 0.47, *p* < 0 0.00) were observed in dogs with coagulopathy. Therefore, care must be taken when managing patients with coagulopathy. The effects of HNPV venom on coagulation factors and renal tissues in this study contrasts with the pronounced effects of elevated PT, aPTT, CT, BUN, creatinine and decreased concertation of fibrinogen. Therefore, it is crucial that all the HNPV bitten dogs are observed and have investigations done to assess the presence of coagulopathy or AKI. Further, CT is a very simple, cost effective bed site test which can be used to detect VICC even in resource poor clinical settings. Accordingly, it is important to note that the more subtle effects of HNPV venom on the coagulation pathway and renal tissues may yet be found by more detailed biochemical analysis since the mechanism of AKI in HNPV envenoming remains unidentified^2^.

Although exact mechanism has yet to be identified, all the HNPV envenomed dogs showed Leukocytosis; which is consistent with *N. naja* bite envenoming ad *D.russelii* bite envenoming in dogs and humans.^21,22,41^. However, the cause of leukocytosis following such snakebite envenoming is yet to be explored^21,22^ and elucidation of this parameter would have the potential to open avenues to estimate the severity of envenoming^22^.Currently available AVS in Sri Lanka is imported from India and uses venom from the Indian species of *Naja. naja, Bangarus caeruleus*, *D. russelii* and *Echis carinatus*. HNPV, which is common in Sri Lanka and South East Asia is not included in the immunization mixtures used in the production of this AVS^42^. Scarcity of AVS has resulted in quantitative measurement of venom concentration within 24 h of snakebite^20^. Therefore, development of antivenom against the deadliest venomous snakes in Sri Lanka must include venom from HNPV to safeguard the life of the patients^43^. When AVS becomes available for HNPV, these assessments will still be beneficial in clinical decision making. Moreover, in depth analysis of venom cytotoxins will be beneficial in exploring refinements to therapeutic intervention for snake envenoming.

## Conclusion

As observed in this study, young male dogs, during dusk and dawn, are the most vulnerable group of HNPV envenoming. The clinical sequelae of HNPV bite envenoming in dogs are varied from local to systemic manifestations. Further, venom of this snake may cause life threatening consequences though death due to HNPV bite envenoming in dogs have not been reported. The currently available Indian antivenom is not applicable in HNPV bite treatment but there is a strong rationale to produce antivenom that can neutralize HNPV venom in order to prevent and control the consequences of envenoming by HNPV.

## Data Availability

The datasets and materials for this study have been retained.
